# 3-Acetyl-6-chloro-4-phenyl­quinolin-2(1*H*)-one

**DOI:** 10.1107/S1600536809054087

**Published:** 2009-12-24

**Authors:** J. Kalyana Sundar, S. Natarajan, S. Sarveswari, V. Vijayakumar, P. L. Nilantha Lakshman

**Affiliations:** a Department of Physics, Madurai Kamaraj University, Madurai 625 021, India; b Organic Chemistry Division, School of Advanced Sciences, VIT University, Vellore 632 014, India; c Department of Food science and Technology, Faculty of Agriculture, University of Ruhuna, Mapalana, Kamburupitiya 81100, Sri Lanka

## Abstract

The title compound, C_17_H_12_ClNO_2_, crystallizes with two mol­ecules in the asymmetric unit. The main conformational difference between these two mol­ecules is the dihedral angle between the phenyl ring and the quinoline ring system [70.5 (1)° and 65.5 (1) Å]. The crystal packing is stabilized by N—H⋯O hydrogen bonds.

## Related literature

For general background, see: Cooper *et al.* (1992 ▶); Gaudio *et al.* (1994 ▶); Gordeev *et al.* (1996 ▶).
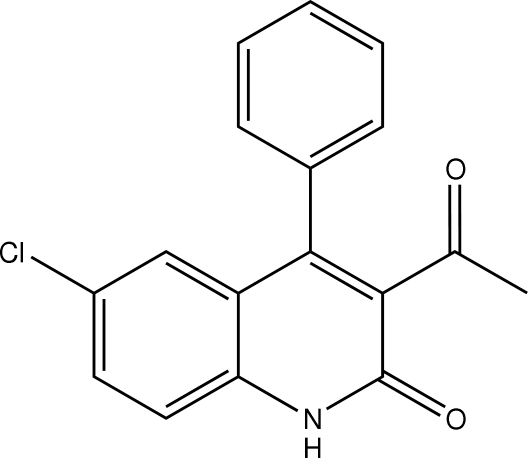


## Experimental

### 

#### Crystal data

C_17_H_12_ClNO_2_
*M* *_r_* = 297.73
Monoclinic, 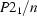

*a* = 10.043 (5) Å
*b* = 18.663 (9) Å
*c* = 15.537 (7) Å
β = 91.811 (5)°
*V* = 2911 (2) Å^3^
*Z* = 8
Mo *K*α radiation
μ = 0.27 mm^−1^
*T* = 293 K
0.17 × 0.14 × 0.11 mm

#### Data collection

Nonius MACH-3 diffractometer
Absorption correction: ψ scan (North *et al.*, 1968 ▶) *T*_min_ = 0.955, *T*_max_ = 0.967
5771 measured reflections
5104 independent reflections
2567 reflections with *I* > 2σ(*I*)
*R*_int_ = 0.015
2 standard reflections every 60 min
intensity decay: none

#### Refinement

*R*[*F* ^2^ > 2σ(*F* ^2^)] = 0.041
*wR*(*F* ^2^) = 0.123
*S* = 1.00
5104 reflections
389 parameters
H atoms treated by a mixture of independent and constrained refinement
Δρ_max_ = 0.22 e Å^−3^
Δρ_min_ = −0.18 e Å^−3^

### 

Data collection: *CAD-4 EXPRESS* (Enraf–Nonius, 1994 ▶); cell refinement: *CAD-4 EXPRESS*; data reduction: *XCAD4* (Harms & Wocadlo, 1996 ▶); program(s) used to solve structure: *SHELXS97* (Sheldrick, 2008 ▶); program(s) used to refine structure: *SHELXL97* (Sheldrick, 2008 ▶); molecular graphics: *PLATON* (Spek, 2009 ▶); software used to prepare material for publication: *SHELXL97*.

## Supplementary Material

Crystal structure: contains datablocks global, I. DOI: 10.1107/S1600536809054087/bt5138sup1.cif

Structure factors: contains datablocks I. DOI: 10.1107/S1600536809054087/bt5138Isup2.hkl

Additional supplementary materials:  crystallographic information; 3D view; checkCIF report

## Figures and Tables

**Table 1:** Hydrogen-bond geometry (Å, °)

*D*—H⋯*A*	*D*—H	H⋯*A*	*D*⋯*A*	*D*—H⋯*A*
N1—H2⋯O4	0.93 (3)	1.88 (3)	2.807 (3)	171 (3)
N2—H1⋯O3	0.96 (3)	1.84 (3)	2.795 (4)	175 (3)

**Fig. 1.**
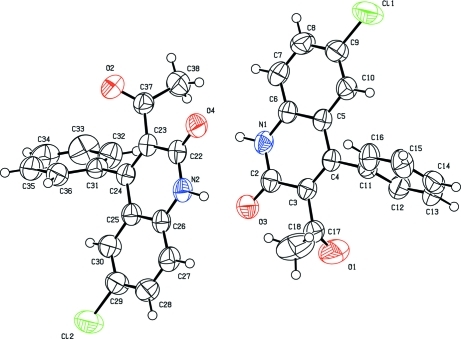
The molecular structure of the title compound, showing 50% probability displacement ellipsoids and the atom-numbering scheme.

## supplementary crystallographic information

### Comment

The molecular structure of the title compound is shown in Fig.1. There are two molecules in the asymmetric unit. The phenyl rings are twisted from the respective quinoline rings by 70.5 (1)° and 65.5 (1) Å.

The two molecules in the asymmetric unit interact through N—H···O bonds (Table 1).

### Experimental

A mixture of 2-amino-5-chlorobenzophenone (2.3 g 0.01 mol) and acetylacetone (1 g, 0.01 mol) with 0.15 ml conc. HCl taken in a beaker was subjected to microwave irradiation for about 6 min. After completion of the reaction (TLC), the reaction mixture was washed with saturated solution NaHCO_3_ (10 ml) and then dried. After that it was washed with petroleum ether and recrystallized with ethanol. m.p.224–226 °C.

### Refinement

The amino H-atom was located in a difference Fourier map, and was freely refined. The C-bound H atoms were placed at calculated positions and allowed to ride on their carrier atoms with C—H = 0.93–0.96 Å and *U*_iso_ = 1.2*U*_eq_(C) for CH group, and 1.5*U*_eq_ for CH_3_ groups.

### Crystal data

**Table:**

C_17_H_12_ClNO_2_	*F*(000) = 1232
*M**_r_* = 297.73	*D*_x_ = 1.359 Mg m^−^^3^
Monoclinic, *P*2_1_/*n*	Mo *K*α radiation, λ = 0.71069 Å
*a* = 10.043 (5) Å	Cell parameters from 25 reflections
*b* = 18.663 (9) Å	θ = 2–25°
*c* = 15.537 (7) Å	µ = 0.27 mm^−^^1^
β = 91.811 (5)°	*T* = 293 K
*V* = 2911 (2) Å^3^	Block, colourless
*Z* = 8	0.17 × 0.14 × 0.11 mm

### Data collection

**Table:**

Nonius MACH-3 diffractometer	2567 reflections with *I* > 2σ(*I*)
Radiation source: fine-focus sealed tube	*R*_int_ = 0.015
graphite	θ_max_ = 25.0°, θ_min_ = 2.2°
ω–2θ scans	*h* = 0→11
Absorption correction: ψ scan (North *et al.*, 1968)	*k* = −1→22
*T*_min_ = 0.955, *T*_max_ = 0.967	*l* = −18→18
5771 measured reflections	2 standard reflections every 60 min
5104 independent reflections	intensity decay: none

### Refinement

**Table:**

Refinement on *F*^2^	Primary atom site location: structure-invariant direct methods
Least-squares matrix: full	Secondary atom site location: difference Fourier map
*R*[*F*^2^ > 2σ(*F*^2^)] = 0.041	Hydrogen site location: inferred from neighbouring sites
*wR*(*F*^2^) = 0.123	H atoms treated by a mixture of independent and constrained refinement
*S* = 1.00	*w* = 1/[σ^2^(*F*_o_^2^) + (0.0478*P*)^2^ + 0.722*P*] where *P* = (*F*_o_^2^ + 2*F*_c_^2^)/3
5104 reflections	(Δ/σ)_max_ < 0.001
389 parameters	Δρ_max_ = 0.22 e Å^−^^3^
0 restraints	Δρ_min_ = −0.18 e Å^−^^3^

### Special details

**Table:**

Geometry. All e.s.d.'s (except the e.s.d. in the dihedral angle between two l.s. planes) are estimated using the full covariance matrix. The cell e.s.d.'s are taken into account individually in the estimation of e.s.d.'s in distances, angles and torsion angles; correlations between e.s.d.'s in cell parameters are only used when they are defined by crystal symmetry. An approximate (isotropic) treatment of cell e.s.d.'s is used for estimating e.s.d.'s involving l.s. planes.
Refinement. Refinement of *F*^2^ against ALL reflections. The weighted *R*-factor *wR* and goodness of fit *S* are based on *F*^2^, conventional *R*-factors *R* are based on *F*, with *F* set to zero for negative *F*^2^. The threshold expression of *F*^2^ > σ(*F*^2^) is used only for calculating *R*-factors(gt) *etc*. and is not relevant to the choice of reflections for refinement. *R*-factors based on *F*^2^ are statistically about twice as large as those based on *F*, and *R*- factors based on ALL data will be even larger.

### Fractional atomic coordinates and isotropic or equivalent isotropic displacement parameters (Å^2^)

**Table:**

	*x*	*y*	*z*	*U*_iso_*/*U*_eq_	
H1	0.316 (3)	0.4766 (17)	0.3548 (19)	0.084 (10)*	
H2	0.433 (3)	0.4103 (15)	0.4718 (18)	0.073 (9)*	
C2	0.2436 (3)	0.38557 (15)	0.48154 (18)	0.0546 (7)	
C3	0.1547 (3)	0.34166 (14)	0.53187 (16)	0.0490 (7)	
C4	0.2019 (3)	0.29962 (14)	0.59716 (16)	0.0478 (7)	
C5	0.3441 (3)	0.29580 (14)	0.61547 (17)	0.0485 (7)	
C6	0.4299 (3)	0.33733 (14)	0.56559 (17)	0.0503 (7)	
C7	0.5675 (3)	0.33402 (16)	0.5801 (2)	0.0637 (8)	
H7	0.6233	0.3615	0.5466	0.076*	
C8	0.6211 (3)	0.29074 (18)	0.6431 (2)	0.0702 (9)	
H8	0.7129	0.2888	0.6528	0.084*	
C9	0.5373 (3)	0.24975 (17)	0.69226 (19)	0.0657 (9)	
C10	0.4021 (3)	0.25169 (16)	0.67917 (18)	0.0583 (8)	
H10	0.3482	0.2234	0.7129	0.070*	
C11	0.1106 (2)	0.25720 (14)	0.65164 (17)	0.0474 (7)	
C12	0.0419 (3)	0.19869 (16)	0.6198 (2)	0.0678 (9)	
H12	0.0509	0.1850	0.5628	0.081*	
C13	−0.0410 (3)	0.16005 (18)	0.6726 (3)	0.0794 (10)	
H13	−0.0875	0.1206	0.6507	0.095*	
C14	−0.0545 (3)	0.17966 (19)	0.7567 (2)	0.0748 (10)	
H14	−0.1102	0.1537	0.7919	0.090*	
C15	0.0141 (3)	0.23736 (19)	0.7888 (2)	0.0763 (10)	
H15	0.0056	0.2505	0.8461	0.092*	
C16	0.0953 (3)	0.27593 (16)	0.7369 (2)	0.0666 (9)	
H16	0.1409	0.3154	0.7593	0.080*	
C17	0.0084 (3)	0.34993 (18)	0.51066 (19)	0.0617 (8)	
C18	−0.0616 (4)	0.4092 (2)	0.5505 (3)	0.1200 (16)	
H18A	−0.1458	0.4165	0.5209	0.180*	
H18B	−0.0090	0.4520	0.5471	0.180*	
H18C	−0.0761	0.3981	0.6098	0.180*	
O3	0.20241 (19)	0.42606 (11)	0.42292 (13)	0.0695 (6)	
C22	0.5109 (3)	0.48911 (15)	0.33411 (18)	0.0540 (7)	
C23	0.6000 (3)	0.51645 (14)	0.26931 (17)	0.0509 (7)	
C24	0.5525 (3)	0.55259 (14)	0.19914 (17)	0.0496 (7)	
C25	0.4099 (3)	0.56463 (15)	0.18733 (17)	0.0516 (7)	
C26	0.3239 (3)	0.53486 (14)	0.24734 (18)	0.0510 (7)	
C27	0.1863 (3)	0.54169 (16)	0.2368 (2)	0.0623 (8)	
H27	0.1307	0.5204	0.2761	0.075*	
C28	0.1325 (3)	0.57966 (17)	0.1688 (2)	0.0680 (9)	
H28	0.0406	0.5846	0.1618	0.082*	
C29	0.2171 (3)	0.61076 (17)	0.11031 (19)	0.0671 (9)	
C30	0.3526 (3)	0.60242 (17)	0.11801 (19)	0.0645 (8)	
H30	0.4068	0.6221	0.0767	0.077*	
C31	0.6424 (3)	0.57753 (16)	0.12980 (17)	0.0527 (7)	
C32	0.7112 (3)	0.52777 (19)	0.0823 (2)	0.0745 (9)	
H32	0.7018	0.4792	0.0938	0.089*	
C33	0.7940 (4)	0.5498 (2)	0.0177 (2)	0.0924 (12)	
H33	0.8403	0.5160	−0.0136	0.111*	
C34	0.8076 (3)	0.6212 (3)	−0.0001 (2)	0.0867 (12)	
H34	0.8627	0.6358	−0.0437	0.104*	
C35	0.7409 (3)	0.6706 (2)	0.0460 (2)	0.0774 (10)	
H35	0.7510	0.7191	0.0342	0.093*	
C36	0.6580 (3)	0.64907 (17)	0.11062 (19)	0.0648 (8)	
H36	0.6121	0.6833	0.1415	0.078*	
C37	0.7460 (3)	0.50148 (17)	0.28466 (17)	0.0565 (8)	
C38	0.7913 (3)	0.42529 (17)	0.2902 (3)	0.0913 (11)	
H38A	0.7661	0.4008	0.2379	0.137*	
H38B	0.7505	0.4023	0.3379	0.137*	
H38C	0.8864	0.4239	0.2983	0.137*	
O4	0.5515 (2)	0.45877 (11)	0.40079 (13)	0.0677 (6)	
N1	0.3760 (2)	0.38020 (13)	0.50155 (15)	0.0564 (6)	
N2	0.3779 (3)	0.49874 (13)	0.31750 (15)	0.0553 (6)	
O1	−0.0477 (3)	0.30921 (15)	0.46303 (19)	0.1134 (9)	
O2	0.8246 (2)	0.55021 (12)	0.28979 (15)	0.0813 (7)	
Cl1	0.60397 (9)	0.19417 (7)	0.77191 (7)	0.1139 (4)	
Cl2	0.15123 (9)	0.65908 (6)	0.02361 (6)	0.0999 (4)	

### Atomic displacement parameters (Å^2^)

**Table:**

	*U*^11^	*U*^22^	*U*^33^	*U*^12^	*U*^13^	*U*^23^
C2	0.060 (2)	0.0565 (19)	0.0479 (17)	−0.0054 (15)	0.0101 (15)	0.0036 (15)
C3	0.0520 (16)	0.0511 (17)	0.0443 (15)	−0.0056 (14)	0.0084 (13)	0.0003 (14)
C4	0.0523 (17)	0.0437 (16)	0.0479 (16)	−0.0037 (13)	0.0105 (13)	−0.0030 (13)
C5	0.0505 (17)	0.0454 (16)	0.0501 (16)	−0.0034 (14)	0.0107 (13)	−0.0038 (14)
C6	0.0542 (18)	0.0470 (16)	0.0501 (17)	−0.0045 (14)	0.0089 (14)	−0.0023 (14)
C7	0.0529 (19)	0.069 (2)	0.070 (2)	−0.0135 (16)	0.0154 (16)	0.0013 (17)
C8	0.0497 (18)	0.085 (2)	0.076 (2)	−0.0036 (18)	0.0037 (16)	0.007 (2)
C9	0.0530 (19)	0.076 (2)	0.069 (2)	0.0034 (16)	0.0095 (16)	0.0154 (17)
C10	0.0526 (18)	0.0593 (19)	0.0640 (19)	−0.0007 (15)	0.0158 (15)	0.0086 (16)
C11	0.0426 (15)	0.0479 (17)	0.0521 (17)	0.0028 (13)	0.0079 (13)	0.0070 (14)
C12	0.068 (2)	0.066 (2)	0.069 (2)	−0.0150 (18)	−0.0003 (16)	0.0054 (17)
C13	0.062 (2)	0.071 (2)	0.104 (3)	−0.0253 (18)	−0.008 (2)	0.023 (2)
C14	0.0497 (19)	0.079 (3)	0.096 (3)	0.0066 (18)	0.0165 (18)	0.040 (2)
C15	0.086 (2)	0.070 (2)	0.075 (2)	0.005 (2)	0.031 (2)	0.0133 (19)
C16	0.075 (2)	0.0580 (19)	0.069 (2)	−0.0048 (16)	0.0233 (17)	0.0036 (16)
C17	0.061 (2)	0.070 (2)	0.0540 (19)	−0.0131 (18)	0.0008 (16)	0.0155 (17)
C18	0.062 (2)	0.148 (4)	0.150 (4)	0.021 (2)	0.007 (2)	−0.056 (3)
O3	0.0667 (13)	0.0785 (15)	0.0636 (13)	−0.0070 (11)	0.0064 (11)	0.0250 (12)
C22	0.065 (2)	0.0456 (17)	0.0517 (19)	−0.0083 (15)	0.0096 (15)	0.0025 (14)
C23	0.0564 (18)	0.0438 (16)	0.0529 (17)	−0.0061 (14)	0.0064 (14)	0.0009 (14)
C24	0.0512 (17)	0.0461 (17)	0.0520 (17)	−0.0047 (13)	0.0084 (14)	−0.0015 (14)
C25	0.0525 (17)	0.0527 (17)	0.0503 (17)	−0.0030 (14)	0.0112 (14)	0.0027 (14)
C26	0.0569 (18)	0.0448 (16)	0.0520 (17)	−0.0033 (14)	0.0137 (14)	0.0016 (14)
C27	0.0556 (19)	0.066 (2)	0.066 (2)	−0.0048 (16)	0.0230 (16)	0.0043 (17)
C28	0.0487 (18)	0.078 (2)	0.078 (2)	0.0020 (17)	0.0134 (17)	0.0088 (19)
C29	0.055 (2)	0.078 (2)	0.069 (2)	0.0055 (16)	0.0119 (16)	0.0189 (17)
C30	0.0538 (19)	0.081 (2)	0.0591 (19)	−0.0009 (16)	0.0145 (15)	0.0191 (17)
C31	0.0457 (16)	0.0646 (19)	0.0481 (16)	−0.0035 (15)	0.0068 (13)	0.0030 (15)
C32	0.071 (2)	0.077 (2)	0.076 (2)	−0.0058 (18)	0.0191 (19)	−0.0045 (19)
C33	0.083 (3)	0.122 (3)	0.074 (2)	0.002 (2)	0.034 (2)	−0.012 (2)
C34	0.057 (2)	0.139 (4)	0.064 (2)	−0.012 (2)	0.0140 (17)	0.021 (2)
C35	0.061 (2)	0.093 (3)	0.079 (2)	−0.0124 (19)	0.0078 (18)	0.029 (2)
C36	0.0572 (19)	0.070 (2)	0.068 (2)	−0.0015 (16)	0.0098 (16)	0.0126 (17)
C37	0.0568 (19)	0.058 (2)	0.0544 (18)	−0.0079 (16)	0.0008 (14)	0.0053 (15)
C38	0.072 (2)	0.064 (2)	0.138 (3)	0.0064 (19)	−0.001 (2)	0.011 (2)
O4	0.0733 (14)	0.0715 (14)	0.0584 (13)	−0.0113 (11)	0.0020 (11)	0.0184 (11)
N1	0.0574 (16)	0.0558 (16)	0.0566 (16)	−0.0119 (13)	0.0096 (13)	0.0084 (13)
N2	0.0586 (17)	0.0535 (15)	0.0546 (15)	−0.0056 (13)	0.0168 (13)	0.0062 (12)
O1	0.099 (2)	0.107 (2)	0.130 (2)	−0.0089 (17)	−0.0444 (17)	−0.0113 (18)
O2	0.0640 (14)	0.0715 (15)	0.1075 (18)	−0.0165 (12)	−0.0092 (13)	0.0025 (13)
Cl1	0.0605 (6)	0.1552 (10)	0.1260 (9)	0.0132 (6)	0.0028 (5)	0.0715 (8)
Cl2	0.0609 (5)	0.1354 (9)	0.1034 (7)	0.0066 (5)	0.0018 (5)	0.0541 (7)

### Geometric parameters (Å, °)

**Table:**

C2—O3	1.244 (3)	C22—N2	1.365 (4)
C2—N1	1.360 (4)	C22—C23	1.460 (4)
C2—C3	1.457 (4)	C23—C24	1.356 (4)
C3—C4	1.356 (3)	C23—C37	1.504 (4)
C3—C17	1.504 (4)	C24—C25	1.455 (4)
C4—C5	1.449 (4)	C24—C31	1.501 (4)
C4—C11	1.494 (3)	C25—C30	1.397 (4)
C5—C10	1.400 (4)	C25—C26	1.405 (4)
C5—C6	1.410 (4)	C26—N2	1.378 (3)
C6—N1	1.374 (3)	C26—C27	1.393 (4)
C6—C7	1.395 (4)	C27—C28	1.369 (4)
C7—C8	1.366 (4)	C27—H27	0.9300
C7—H7	0.9300	C28—C29	1.390 (4)
C8—C9	1.385 (4)	C28—H28	0.9300
C8—H8	0.9300	C29—C30	1.371 (4)
C9—C10	1.367 (4)	C29—Cl2	1.735 (3)
C9—Cl1	1.733 (3)	C30—H30	0.9300
C10—H10	0.9300	C31—C36	1.378 (4)
C11—C12	1.375 (4)	C31—C32	1.385 (4)
C11—C16	1.383 (4)	C32—C33	1.385 (4)
C12—C13	1.389 (4)	C32—H32	0.9300
C12—H12	0.9300	C33—C34	1.368 (5)
C13—C14	1.368 (5)	C33—H33	0.9300
C13—H13	0.9300	C34—C35	1.358 (5)
C14—C15	1.364 (4)	C34—H34	0.9300
C14—H14	0.9300	C35—C36	1.384 (4)
C15—C16	1.370 (4)	C35—H35	0.9300
C15—H15	0.9300	C36—H36	0.9300
C16—H16	0.9300	C37—O2	1.206 (3)
C17—O1	1.190 (3)	C37—C38	1.495 (4)
C17—C18	1.459 (5)	C38—H38A	0.9600
C18—H18A	0.9600	C38—H38B	0.9600
C18—H18B	0.9600	C38—H38C	0.9600
C18—H18C	0.9600	N1—H2	0.93 (3)
C22—O4	1.238 (3)	N2—H1	0.96 (3)
			
O3—C2—N1	120.8 (3)	C24—C23—C22	121.4 (3)
O3—C2—C3	122.6 (3)	C24—C23—C37	122.5 (2)
N1—C2—C3	116.5 (3)	C22—C23—C37	116.2 (3)
C4—C3—C2	121.4 (3)	C23—C24—C25	119.9 (2)
C4—C3—C17	122.6 (2)	C23—C24—C31	121.8 (2)
C2—C3—C17	115.8 (2)	C25—C24—C31	118.3 (2)
C3—C4—C5	119.6 (2)	C30—C25—C26	117.7 (3)
C3—C4—C11	121.6 (2)	C30—C25—C24	123.7 (2)
C5—C4—C11	118.8 (2)	C26—C25—C24	118.6 (3)
C10—C5—C6	117.6 (3)	N2—C26—C27	120.1 (2)
C10—C5—C4	123.6 (2)	N2—C26—C25	118.9 (3)
C6—C5—C4	118.7 (3)	C27—C26—C25	120.9 (3)
N1—C6—C7	120.6 (3)	C28—C27—C26	120.3 (3)
N1—C6—C5	119.0 (3)	C28—C27—H27	119.9
C7—C6—C5	120.5 (3)	C26—C27—H27	119.9
C8—C7—C6	120.5 (3)	C27—C28—C29	119.1 (3)
C8—C7—H7	119.8	C27—C28—H28	120.5
C6—C7—H7	119.8	C29—C28—H28	120.5
C7—C8—C9	119.3 (3)	C30—C29—C28	121.4 (3)
C7—C8—H8	120.3	C30—C29—Cl2	118.7 (2)
C9—C8—H8	120.3	C28—C29—Cl2	119.9 (2)
C10—C9—C8	121.4 (3)	C29—C30—C25	120.6 (3)
C10—C9—Cl1	118.8 (2)	C29—C30—H30	119.7
C8—C9—Cl1	119.8 (2)	C25—C30—H30	119.7
C9—C10—C5	120.7 (3)	C36—C31—C32	118.2 (3)
C9—C10—H10	119.7	C36—C31—C24	122.0 (3)
C5—C10—H10	119.7	C32—C31—C24	119.8 (3)
C12—C11—C16	118.4 (3)	C31—C32—C33	120.5 (3)
C12—C11—C4	121.8 (3)	C31—C32—H32	119.7
C16—C11—C4	119.8 (2)	C33—C32—H32	119.7
C11—C12—C13	120.2 (3)	C34—C33—C32	120.1 (3)
C11—C12—H12	119.9	C34—C33—H33	120.0
C13—C12—H12	119.9	C32—C33—H33	120.0
C14—C13—C12	120.3 (3)	C35—C34—C33	120.1 (3)
C14—C13—H13	119.8	C35—C34—H34	120.0
C12—C13—H13	119.8	C33—C34—H34	120.0
C15—C14—C13	119.8 (3)	C34—C35—C36	120.2 (3)
C15—C14—H14	120.1	C34—C35—H35	119.9
C13—C14—H14	120.1	C36—C35—H35	119.9
C14—C15—C16	120.2 (3)	C31—C36—C35	120.9 (3)
C14—C15—H15	119.9	C31—C36—H36	119.6
C16—C15—H15	119.9	C35—C36—H36	119.6
C15—C16—C11	121.2 (3)	O2—C37—C38	121.1 (3)
C15—C16—H16	119.4	O2—C37—C23	120.2 (3)
C11—C16—H16	119.4	C38—C37—C23	118.7 (3)
O1—C17—C18	121.5 (3)	C37—C38—H38A	109.5
O1—C17—C3	120.7 (3)	C37—C38—H38B	109.5
C18—C17—C3	117.7 (3)	H38A—C38—H38B	109.5
C17—C18—H18A	109.5	C37—C38—H38C	109.5
C17—C18—H18B	109.5	H38A—C38—H38C	109.5
H18A—C18—H18B	109.5	H38B—C38—H38C	109.5
C17—C18—H18C	109.5	C2—N1—C6	124.6 (2)
H18A—C18—H18C	109.5	C2—N1—H2	116.8 (18)
H18B—C18—H18C	109.5	C6—N1—H2	118.4 (18)
O4—C22—N2	120.9 (2)	C22—N2—C26	124.9 (2)
O4—C22—C23	123.0 (3)	C22—N2—H1	118.6 (18)
N2—C22—C23	116.2 (3)	C26—N2—H1	116.4 (18)
			
O3—C2—C3—C4	−178.0 (3)	C22—C23—C24—C25	−0.1 (4)
N1—C2—C3—C4	2.2 (4)	C37—C23—C24—C25	179.2 (3)
O3—C2—C3—C17	−1.9 (4)	C22—C23—C24—C31	−176.7 (2)
N1—C2—C3—C17	178.2 (3)	C37—C23—C24—C31	2.6 (4)
C2—C3—C4—C5	−2.5 (4)	C23—C24—C25—C30	178.7 (3)
C17—C3—C4—C5	−178.2 (3)	C31—C24—C25—C30	−4.6 (4)
C2—C3—C4—C11	177.0 (2)	C23—C24—C25—C26	−3.3 (4)
C17—C3—C4—C11	1.3 (4)	C31—C24—C25—C26	173.4 (2)
C3—C4—C5—C10	−177.3 (3)	C30—C25—C26—N2	−178.5 (2)
C11—C4—C5—C10	3.2 (4)	C24—C25—C26—N2	3.4 (4)
C3—C4—C5—C6	1.3 (4)	C30—C25—C26—C27	1.3 (4)
C11—C4—C5—C6	−178.3 (2)	C24—C25—C26—C27	−176.8 (3)
C10—C5—C6—N1	178.9 (2)	N2—C26—C27—C28	177.7 (3)
C4—C5—C6—N1	0.2 (4)	C25—C26—C27—C28	−2.0 (4)
C10—C5—C6—C7	−0.1 (4)	C26—C27—C28—C29	0.4 (5)
C4—C5—C6—C7	−178.7 (2)	C27—C28—C29—C30	1.9 (5)
N1—C6—C7—C8	−179.2 (3)	C27—C28—C29—Cl2	180.0 (2)
C5—C6—C7—C8	−0.3 (4)	C28—C29—C30—C25	−2.7 (5)
C6—C7—C8—C9	0.4 (5)	Cl2—C29—C30—C25	179.3 (2)
C7—C8—C9—C10	0.0 (5)	C26—C25—C30—C29	1.0 (4)
C7—C8—C9—Cl1	179.6 (2)	C24—C25—C30—C29	179.0 (3)
C8—C9—C10—C5	−0.3 (5)	C23—C24—C31—C36	−117.9 (3)
Cl1—C9—C10—C5	−180.0 (2)	C25—C24—C31—C36	65.5 (4)
C6—C5—C10—C9	0.4 (4)	C23—C24—C31—C32	63.0 (4)
C4—C5—C10—C9	178.9 (3)	C25—C24—C31—C32	−113.7 (3)
C3—C4—C11—C12	70.2 (4)	C36—C31—C32—C33	0.4 (5)
C5—C4—C11—C12	−110.3 (3)	C24—C31—C32—C33	179.6 (3)
C3—C4—C11—C16	−110.8 (3)	C31—C32—C33—C34	−0.5 (5)
C5—C4—C11—C16	68.7 (3)	C32—C33—C34—C35	0.5 (6)
C16—C11—C12—C13	0.3 (4)	C33—C34—C35—C36	−0.6 (5)
C4—C11—C12—C13	179.3 (3)	C32—C31—C36—C35	−0.5 (4)
C11—C12—C13—C14	−0.3 (5)	C24—C31—C36—C35	−179.6 (3)
C12—C13—C14—C15	−0.2 (5)	C34—C35—C36—C31	0.5 (5)
C13—C14—C15—C16	0.6 (5)	C24—C23—C37—O2	58.7 (4)
C14—C15—C16—C11	−0.6 (5)	C22—C23—C37—O2	−122.0 (3)
C12—C11—C16—C15	0.2 (4)	C24—C23—C37—C38	−119.2 (3)
C4—C11—C16—C15	−178.9 (3)	C22—C23—C37—C38	60.2 (4)
C4—C3—C17—O1	−87.4 (4)	O3—C2—N1—C6	179.5 (3)
C2—C3—C17—O1	96.6 (4)	C3—C2—N1—C6	−0.7 (4)
C4—C3—C17—C18	92.4 (4)	C7—C6—N1—C2	178.4 (3)
C2—C3—C17—C18	−83.5 (4)	C5—C6—N1—C2	−0.5 (4)
O4—C22—C23—C24	−176.6 (3)	O4—C22—N2—C26	176.7 (3)
N2—C22—C23—C24	3.4 (4)	C23—C22—N2—C26	−3.3 (4)
O4—C22—C23—C37	4.0 (4)	C27—C26—N2—C22	−179.8 (3)
N2—C22—C23—C37	−176.0 (2)	C25—C26—N2—C22	0.0 (4)

### Hydrogen-bond geometry (Å, °)

**Table:**

*D*—H···*A*	*D*—H	H···*A*	*D*···*A*	*D*—H···*A*
N1—H2···O4	0.93 (3)	1.88 (3)	2.807 (3)	171 (3)
N2—H1···O3	0.96 (3)	1.84 (3)	2.795 (4)	175 (3)
